# Subsets of the population benefitting from the pandemic: What policies and practices should be arranged to sustainably maintain beneficiaries' mental health

**DOI:** 10.3389/fsoc.2022.1090785

**Published:** 2022-12-15

**Authors:** Jason Hung

**Affiliations:** Department of Sociology, University of Cambridge, Cambridge, United Kingdom

**Keywords:** mental health, pandemic, COVID-19, digitalisation, public policies

## Abstract

Here researchers have the disposition to engage in the scholarly discourse on how the pandemic adversely influenced individuals' mental health and what remedies should be exercised in response to the mental health challenges. There is a shortage of scholarly discussion about who benefitted from the occurrence of the pandemic. Mancini et al. argued that the pandemic benefitted the social and mental health functioning of a subset of the population, despite the pandemic causing considerable risks of harm to mental health. In this perspective, the author summarizes relevant findings and arguments to present which subsets of the population benefitted at school, at home, and in the workplace during the pandemic. Although COVID-19 is no longer deemed a pandemic, many by-products of the public health crisis, including the encouragement of remote work and studies, remain. In this perspective, by understanding who benefitted from the pandemic and why, the author can evaluate if any public policies formed in response to the pandemic should be kept in the long run in order to maximize individuals' mental health.

## 1. Introduction

Since the COVID-19 pandemic, a raft of studies has been developed to examine the nuanced relationships between the pandemic and mental health at local, regional and global levels. However, relevant literature predominantly focused on assessing to what extent the pandemic and the associated consequences, such as the application of social distancing rules and home quarantine measures, negatively impacted individuals' mental health (Cullen et al., 2020; Pfefferbaum and North, 2020). The remaining studies primarily addressed how individuals' mental health could improve during the pandemic (Callow et al., 2020; Bowe et al., 2022). Here researchers have the disposition to engage in the scholarly discourse on how the pandemic adversely influenced individuals' mental health and what remedies should be exercised in response to the mental health challenges. There is a shortage of scholarly discussion about who benefitted from the occurrence of the pandemic. Mancini (2020) argued that the pandemic benefitted the social and mental health functioning of a subset of the population, despite the pandemic causing considerable risks of harm to mental health. In this perspective, the author summarizes relevant findings and arguments to present which subsets of the population benefitted at school, at home, and in the workplace during the pandemic. Although COVID-19 is no longer deemed a pandemic, many by-products of the public health crisis, including the encouragement of remote work and studies, remain. In this perspective, by understanding who benefitted from the pandemic and why, the author can evaluate if any public policies formed in response to the pandemic should be kept in the long run in order to maximize individuals' mental health.

## 2. At school

In a Chinese survey carried out March 13–20, 2020, a total of 4,391 primary and secondary school students reported their wellbeing (Tang et al., 2021). Findings suggested that Chinese students who had stronger and more positive family ties prior to the outbreak of the pandemic were prone to displaying more desirable levels of life satisfaction during the lockdowns and home confinement. In addition, students who perceived home confinement as beneficial, owing to the increase in family time and allowance for personal activities, reported more life satisfaction (Tang et al., 2021). An additional supporting American study showed that school-aged students enjoyed the comfort of spending quality time with families and the absence of assuming non-domestic responsibilities amid the pandemic. This circumstance heightened their capacity to make time for workout, spend time on hobbies and focus primarily on studies. These advantages improved the mental health of these school-aged students (Schlesselman et al., 2020).

A more comprehensive British study was developed using data collected in June and July 2020 from the OxWell Student Survey (Soneson et al., 2021). Some one-third (i.e., 33.2%) of British students revealed that their mental health improved during lockdowns. In line with the findings from Tang et al. (2021), Soneson et al. (2021) found that British students with better family relationships purportedly enjoyed better mental health during lockdowns. Moreover, British students who were victimized by school bullying and poor relationships with teachers had better mental health during home quarantines (Soneson et al., 2021). British students justified how a healthy relationship with the household was conducive to personal wellbeing by noting that they felt safe at home (Soneson et al., 2021). Moreover, when the United States was in lockdown during the first local wave of the COVID-19, remote schooling in lieu of on-campus schooling was applied. Here Hicks et al. (2021) discovered that American high school students nationwide reportedly experienced some 30–35 percent drop in school bullying and cyberbullying during the course. Those who benefitted from the decrease in bullying victimization demonstrated a significant improvement in their mental health (Hicks et al., 2021).

While the pandemic ceased and on-campus schooling has resumed in many countries, there has been a growing number of children and adolescents being permanently educated at home in the West since the outbreak of the pandemic. For example, the number of parents deciding to homeschool their children has risen by 34% in England alone between February 2020 and February 2022 (Increase in Home, 2022). The public health crisis and the associated need to adopt homeschooling or working from home offered individuals opportunities to experience the benefits of performing activities domestically. Such a circumstance has encouraged the growth of homeschooling in the post-pandemic epoch.

For those who are subject to remote schooling, parents and guardians should serve as the primary stakeholders in monitoring their wellbeing and academic performance. In the United Kingdom, for example, parents or guardians are allowed to let children and adolescents receive home education. Parents or guardians contact local councils, and representatives will check if children and adolescents can possibly receive a suitable education at home. If local council representatives evaluate the enquiries from parents or guardians and decide that homeschooling is inappropriate for the children or adolescents, a school attendance order (SAO) will be issued. SAOs are issued by local council representatives if they find that any children or adolescents are not getting an appropriate education. Parents or guardians receiving an SAO are given 15 days to provide evidence that the children or adolescents register with a school listed in the SAO or that an appropriate home education setting has been arranged (GOV.UK, 2022). Other countries should follow the practice of the United Kingdom and require all parents or guardians who want children or adolescents to be homeschooled to demonstrate to a local council that home education materials, resources, and facilities have been aptly prepared. Parents or guardians who homeschool children and adolescents but fail to provide the necessary home education setting should be legally sanctioned. Such a practice should ensure that no children are deprived of the right to sufficient education, regardless of whether homeschooling or on-campus learning applies.

The findings of an additional study carried out by Silk et al. (2022) echoed Tang et al. (2021) and Soneson et al. (2021), in noting that 20 and 14% of Canadian students reportedly suffered from less depression and anxiety symptoms, respectively, after the pandemic (Silk et al., 2022). The primary reasons are the avoidance of peer victimization, the entitlement to more family and personal leisure time, sleeping more sufficiently, and the experience of less academic stress. Canadian adolescents spent far less time on schoolwork when they were subject to remote learning and could have better control over their own schedules (Increase in Home, 2022). Again, the mental health of students who have better time management and self-discipline can improve insofar as they remain academically independent.

Parents and guardians who are deciding to homeschool their children might prefer sending them to school in childhood and early adolescence whilst allowing remote learning when they enter the later stage of adolescence. Such a strategy enables students to develop personal skills in time management and self-discipline strategically at school (Tereshchuk, 2021; Morse et al., 2022). When they become more academically independent as they grow, homeschooling may serve as a beneficial and appropriate alternative. For those who request and receive approval, homeschooling children or adolescents at local councils or their equivalents, the children's or adolescents' records should be transferred to the local social welfare departments or their equivalents (GOV.UK, 2022). Each homeschooled student should be assigned a social worker who should maintain regular communication with each student. Social workers can share some of the responsibilities of the youth's parents and check the social, academic, and psychological wellbeing of the youth on a regular basis (Rothermel, 2012). Moreover, social workers serve as reliable and trustable figures to whom youth with mental health challenges can reach out and seek advice where appropriate.

While homeschooling can minimize peer victimization, children and adolescents are exposed to higher risks of cyberbullying, primarily owing to their increased engagement in social media (Armitage, 2021). Armitage argued that the prevalence of cyberbullying is far lower than that of conventional forms of bullying (Armitage, 2021). However, cyberbullying victimization is detrimental. Victims suffer from poor mental health. Social workers should actively check if students are spending more time online and experiencing any form of cyberbullying that jeopardizes their mental health. If so, they should communicate with students' parents or guardians to arrange better family support. Parents and guardians may also encourage children and adolescents to visit general practitioners regularly, if the student's wellbeing is observably unstable or negative.

## 3. At home

Those who school or work remotely, they primarily spend their time domestically. Researchers from Cardiff University and Cardiff Metropolitan University found that individuals living in any setting with a private or shared garden or a park within the neighborhood were calmer and more peaceful and felt more energized during the pandemic (Poortinga, 2021). They further claimed that access to green space was pivotal to the mental health of individuals who were predominantly kept at home (Poortinga, 2021). However, in the United Kingdom, for example, one in eight households had no access to a private or shared garden (Poortinga, 2021). In densely populated regions where the practice of urban planning is significantly different from the United Kingdom, such as metropolitan cities in East and Southeast Asia, having a private or shared garden is almost luxuriously unreachable. An additional study was carried out with 323 students (average age: 21.99 years-old) in Plovdiv, Bulgaria from 17th May 2020 to 10th June 2020. Findings showed that respondents who were confined at home during the pandemic enjoyed reduced anxiety and depression symptoms if they could see green spaces within their homes or in their neighborhoods. Conclusions were made that students spending the majority of their time in home quarantine experienced better mental health if they were exposed to more greenery (Dzhambov et al., 2021). Along with greenery, a Chicago survey unveiled that home-quarantined residents who were able to see wildlife generally enjoyed better physical and mental health. The privilege to visibly access greenery and wildlife helped mitigate any negative emotions imposed by prolonged home-quarantine, leading to better wellbeing (Murray et al., 2022).

As homeschooling and remote working become increasingly popular, more densely populated metropolitan cities should accelerate their practice of green urban planning. Prior to and after the pandemic, Singapore has been known as one of the pioneers of exercising vertical greening, in which a growth of buildings has been designed and built with the presence of green spaces (Simmons, 2022). More metropolitan governments should follow Singapore and create more green spaces in condominiums and apartments. Such an approach enables residents to gain access to green environments in order to mitigate their encounters with mental health stressors.

## 4. At work

Along with residential establishments, vertical greening should be applied to the construction of commercial buildings, facilitating employee access to green spaces. For employees who work in hybrid or completely remote modes, better mental health results when companies set up a clear code of conduct designated for supervisors to monitor the wellbeing of their supervisees (CIPD, 2022). Supervisors should check in with their supervisees individually on a regular basis. This is an opportunity for supervisors to observe any signals of poor mental health from their supervisees. If any signals are alerted, supervisors should arrange a wellbeing conversation with the supervisees. Representatives from the human resources departments have to ensure supervisors have a clear process to follow up on the wellbeing discourse. These suggestions are supported by a survey conducted in April 2020 in the United States. Survey findings indicated that in order to improve employees' mental health during the public health crisis, it is the supervisor's responsibilities to adapt a supportive role by maintaining regular supervisor-supervisee communications in order to encourage employees to receive constructive advice on how to juggle work and non-work issues (Evanoff et al., 2020).

Also, insofar as hybrid or remote working is permitted, supervisors should hold regular virtual events. These events help maintain and strengthen the social connections between team members, especially when within-team collaboration is significantly required as part of the job nature (CIPD, 2022). As employees who experience mental health challenges might prefer the reduction of social contact with others, virtual or not, participation in these virtual events arranged by the supervisors should be optional. It is noteworthy that Byrne et al. (2021) argued work units who were able to provide virtual well-being support and deliver the provision of rapidly accessible mental health professionals to virtually screen and virtually assess employees' mental health would facilitate the early detection of COVID-19-related stressors that caused them to suffer from dissatisfactory mental health. These professionals could also provide mental health intervention to employees on the verge of developing alarming mental health issues. Such a e-service helped employees receive necessary mental health support and ensure all employees within the work units were entitled to sustainable, better wellbeing (Byrne et al., 2021).

## Conclusion

Due to the increasing digitalisation and the pandemic, homeschooling and remote working have been practiced more often or permanently among some subsets of the population. The relationships between mental health and associated consequences derived from the pandemic are nuanced. While home education and remote work are in favor of some cohorts' mental health, these digitalised, online practices can be detrimental to the rest. At the family and workplace levels, parents, guardians or supervisors should assume their responsibilities to monitor the wellbeing of those who engage in online studies or work. At the community level, additionally, government-run or government-affiliated units should provide necessary social support to children, adolescents and even adults who experience any mental health issues imposed by online activities. In developing countries where the social burdens on the distribution of welfare and benefits are unmanageable, government-run and government-affiliated units should be subsidized by the central governments to provide financial incentives to improve human resources retention. Volunteers should also be publicly recruited within the communities. Ensuring the availability of sufficient human resources helps specialists and volunteers deliver adequate remote social support to those who encounter mental health issues caused by the pandemic. These support services can be offered via telecommunications and social media platforms, in order to maintain social distancing measures and facilitate convenience. At the government level, government officials should establish laws and regulations that require all (prospective) homeschooled students to register at local government units. Representatives from the units should evaluate if homeschooling is feasible and sustainable on a case-by-case basis. Government officials should also facilitate their green urban planning, for the purpose of establishing more green spaces that are accessible to everyone indiscriminatingly.

## Data availability statement

The original contributions presented in the study are included in the article/supplementary material, further inquiries can be directed to the corresponding author.

## Author contributions

JH is solely responsible for designing, writing, reviewing, editing, and submitting this manuscript for publication consideration.
